# Postnatal care utilization and associated factors among women who gave birth within the last 12 months in northwest Ethiopia: a cross-sectional study

**DOI:** 10.11604/pamj.2024.48.126.42397

**Published:** 2024-07-24

**Authors:** Tesfahun Hailemariam, Asmamaw Atnafu, Lemma Derseh Gezie, Teshale Belayneh, Binyam Tilahun

**Affiliations:** 1 Department of Health Informatics, Institute of Public Health, College of Medicine and Health Sciences, University of Gondar, Gondar, Ethiopia,; 2 Department of Health System and Policy, Institute of Public Health, College of Medicine and Health Sciences, University of Gondar, Gondar, Ethiopia,; 3 Department of Epidemiology and Biostatistics, Institute of Public Health, College of Medicine and Health Sciences, University of Gondar, Gondar, Ethiopia,; 4 Department of Public Health, Hawassa College of Health Sciences, Hawassa, Ethiopia

**Keywords:** Postnatal care, women’s education, healthcare accessibility, northwest, Ethiopia

## Abstract

**Introduction:**

postnatal care after birth is a crucial component in saving the lives of mothers and new-borns. A paucity of evidence indicated that women's unwillingness to receive care after birth remains a challenge in resource-limited settings in general and in Ethiopia in particular. This study aimed to assess the level of postnatal care utilization and factors affecting it in northwest Ethiopia.

**Methods:**

a community-based cross-sectional study was conducted from October to November 2020. A total of 811 women who had given birth within the last year were involved in the study. Both random and fixed effects were reported using an adjusted odds ratio with a 95% confidence interval and a p-value of <0.05.

**Results:**

the study revealed that postnatal care utilization was 19.85% (95% CI: 20.8-29.4) in northwest Ethiopia. Maternal education of secondary and above (AOR =2.65; 95% CI: 1.43, 4.94); facility delivery (AOR = 3.99; 95% CI: 2.44, 6.55); membership in women associations in the community (AOR = 1.6; 95% CI: 1.07, 2.4); four or more ANC contacts (AOR = 1.51; 95% CI: 1.03, 2.22); having health education at health post level (AOR = 1.77; 95% CI: 1.21, 2.58), and birth order (AOR = 2.64; 95% CI: 1.21, 5.78) were significantly associated with postnatal care utilization.

**Conclusion:**

postnatal care utilization is low in rural northwest Ethiopia compared to findings from most resource-limited settings. The existing health system should consider community-based intervention strategies focusing on maternal health education, healthcare accessibility, and promoting women's participation in the community to be more effective in improving postnatal care utilization.

## Introduction

The postnatal period includes the period from the birth of the baby and extends up to six weeks, or 42 days [1]. The postnatal period is a critical stage in the lives of mothers and new-borns [2]. Most maternal and infant deaths occur during this time, yet this is the most neglected period for the provision of quality care [1,2]. Globally, the Maternal Mortality Ratio (MMR) was 223 deaths per 100,000 live births in 2020 [3]. Worldwide, over 65% of maternal deaths occur during the first 42 days of postpartum, with the same amount of neonatal deaths occurring during the first 7 days of their lives [4]. Almost 95% of all maternal deaths occurred in low and lower middle-income countries, and most could have been prevented [3]. In sub-Saharan Africa, pregnancy-related and new-born mortalities are arguably high. The MMR in the African Region was 531 deaths per 100,000 live births, accounting for 69% of global maternal deaths in 2020 [5]. The magnitude of postnatal care utilization in sub-Saharan African countries was 52.48%, with the highest postnatal care utilization in the Central Region of Africa (73.51%) and the lowest postnatal care utilization in the eastern Regions of Africa (31.71%) [6].

In Ethiopia, women receive at least one postnatal care service within the first 48 hours after birth, which is only 17%, which is very low even in comparison with the other regions of sub-Saharan African countries where its coverage reaches from 31.7% to 52.5% [7,8]. A large proportion of maternal deaths occur during the first 48 hours after birth [9]. Thus, receiving Postnatal Care (PNC) from a healthcare provider at the recommended time with its appropriate contents also prevents complications that could arise after childbirth [10]. About 71% of rural women do not receive a postnatal check-up within 2 days of delivery, whereas 52% of urban women do not receive an early postnatal check-up [9]. From the previous studies, postnatal care service utilization is affected by many factors throughout the world, including women's educational status, age, occupation, place of birth, PNC service awareness, Antenatal Care (ANC) follow-up, place of residence, parity, household wealth index, birth order, skilled birth attendance during delivery, media exposure, caesarean delivery, having good knowledge of PNC and being a model household [6, 11-18]. However, there is a significant variation in risk factors for postnatal care utilization in sub-Saharan African countries [19,20]. Our study findings will contribute to designing an effective program to the reduce risk factors of postnatal care service non-utilization. It also helps program managers, policymakers, and service providers for women and new-borns after birth. Despite this fact, early PNC service use plays a critical role in reducing maternal and new-borns mortality. The proportion of postnatal care service utilization is very low, and much is not known about its determinant factors in Ethiopia. This study aimed to determine the prevalence of early postnatal care service use and associated factors among mothers who had given birth in the last 12 months.

## Methods

**Study area and period**: this study was conducted in two districts of the Central Gondar Zone, northwest Ethiopia. The districts are located 658km from Addis Ababa, the capital city of Ethiopia. There were 16 health centers and 88 health posts in the districts. The total population was 524,907 (female = 26,0879 and male = 264,228) at the time of the survey, of which 122,303 and 16,745 were women in the reproductive age group and surviving infants, respectively. The study period of this study was from October to November 2020.

**Study design**: a community-based cross-sectional study was conducted.

**Source population**: all lactating women who gave birth within the past 12 months and lived in the study districts were the target population.

**Study population**: lactating women who gave birth within the past 12 months and were permanent residents of the selected *kebeles* were the study population.

**Sample size determination**: both the single population proportion and the double population proportion formulas were considered in estimating different sample sizes. For the single population proportion formula, the assumptions used were: prevalence of ANC attendance [21], prevalence of completion of four or more antenatal care visits [21], the proportion of mothers who received delivery service from health care providers after completing antenatal care four and above, and the proportion of women retained in the continuum of maternal care [21], considering a 95% CI, a margin of error of 5%, design effect of 2, and a 10% non-response rate. For double population proportion formulas, the sample size was computed using the STAT-CALC program of Epi-Info version 7.0. software using the following assumptions: 5% level of significance (two-sided), 80% power, and a 1:1 ratio of exposed to non-exposed and odds ratio. Factors such as being a model household in the community [22], distance to reach a health facility [23], and antenatal care visits [24] were considered predictor variables. The design effect of 2 and 10% non-response rate were used for sample size estimations, respectively. Of the different sample sizes estimated, the maximum sample size, 811, was obtained from a single population formula and considered in the study.

**Sampling procedure**: a multi-stage cluster sampling technique was employed to reach study participants. Wogera and Gondar Zuriya districts were selected randomly among the six transformation *woredas* in the central Gondar zone. Specifically, the primary sampling units were *kebeles* with the respective health centers, while the secondary sampling units were women who gave birth during the past 12 months. The health centers (or the respective *kebeles*) were selected with a simple random sampling technique. To select sample participants, a sampling frame of mothers was developed using a list of eligible women from the community health information system family folder (pouch). Then the sampling interval was obtained by dividing the source population (8393) by the estimated total sample size (811). The first study participant (the 2^nd^ mother) was selected by a simple random sampling technique from the first sampling interval, and all other mothers were selected systematically by taking every 10^th^ mother in the frame. When two or more women were found at a single household level, one of them was selected randomly and included in the study. Seriously ill women or women who were unable to speak were excluded from the study. For those women who were selected for an interview but were not available during data collection, we waited for them for three to five days to get them. If they were not still accessible, they would be considered non-respondents.

### Variables and measurement

**Outcome variables**: the outcome variable of this study was postnatal care utilization. Postnatal care utilization was classified as "yes" if a woman received postnatal care utilization after birth, or "no" if otherwise.

**Explanatory variables**: explanatory variables were the age of the respondents, education status of respondents, education status of partner, intendedness of the current pregnancy, being a member of community-based health insurance, mass-media exposure, and health extension workers' home visits during pregnancy, being a member of the women's development army, parity, access to transportation, and household wealth index. For wealth index assessment, principal component analysis was employed to reduce data that measure the same construct together, and the data were recoded into binary variables. The wealth quantile was categorized into five categories: poorest, poorer, middle, richer, and richest.

**Data collection procedures and tools**: an interviewer-administered questionnaire was used to collect the household data. The tool was prepared in English after a thorough literature review, translated into the local language (Amharic), and then finally returned to English. Since experts' views were sought for the psychometric properties of the face and content validity, experts were invited to review the relevance of each question in the tool. The tool was refined according to the expert view, piloted out of the study setting, and validated before data collection. The proportions of the rated item content validity index and scale content validity index were 0.98 and 0.81, respectively. Six data collectors and two supervisors were recruited for data collection and supervision. The data were collected using an interviewer-administered questionnaire via face-to-face interview.

**Data quality assurance**: two-day training was given to data collectors and supervisors regarding the objective of the study, procedures for data collection, data collection tools, and handling of the data. During the data collection period, daily supervision was conducted by the supervisors.

**Data management and statistical analysis**: data were entered into EpiData 4.02 and then exported to STATA 14 software for statistical analysis. Principal Component Analysis (PCA) was constructed to assess household wealth status, and the items were recoded into two categories (0 = no, 1 = yes) to reduce the amount of data that measures the same construct. Twenty-one items composed of household productive and non-productive assets and utilities were entered for analysis. The frequencies of the assets greater than 95% and less than 5% were excluded from the analysis as it would not help to identify the richer or poorer category. The scale reliability coefficient and the Kaiser-Meyer-Olkin measure of sampling adequacy were employed to assess the satisfaction of the assumptions for PCA. The wealth quantile was categorized into five categories: poorest, poorer, middle, richer, and richest. To identify factors associated with response variables, a bi-variable logistic regression analysis was fitted. Likewise, the model’s fitness was checked using the Hosmer and Lemeshow goodness of fitness tests. The correlation of independent variables was checked by using the Variance Inflation Factor (VIF), in which VIF > 10 indicates substantial multicollinearity, and there was no multicollinearity effect among predictor variables. Descriptive statistics such as frequency and cross-tabulation were computed. In the bivariable logistic regression model, a p < 0.25 was used to identify candidate variables for multivariable model, and the selected variables were entered sequentially by using backward stepwise regression. An association between outcome variables and explanatory variables was presented using an adjusted OR with a 95% confidence interval. A significance level of p < 0.05 was considered to indicate the strength of association with women's optimal antenatal care visits and institutional delivery.

**Ethical considerations**: study approval and ethical clearance were obtained from the University of Gondar ethical review board (R.NO. V/P/RCS/05/2020). A formal letter of approval was taken from the Amhara National Regional Health State Bureau and the Central Gondar Zonal Health Department. Written informed consent was obtained from the participants and their parents, and assent was obtained from the minor/participant. They were informed about the objective, importance of the study, procedure and duration, risk and discomfort, benefits of participating in the study, confidentiality, and the right to refuse or withdraw during data collection. After obtaining the relevant information, participants were counselled on the benefits of attending maternal healthcare services and the consequences of missing maternal healthcare services. All methods were carried out in accordance with relevant guidelines and regulations.

## Results

**Sociodemographic characteristics of the study participants**: a total of 811 women participated in this study, with a 100% response rate. The median age of the participants was 28 years (+10), with an interquartile range of 23 to 33 years. The proportion of women who had attended secondary education and above was 96 (11.8%). The proportion of women was nearly equal across the wealth quantiles of the household level, with the poorest 163 (20.1%), poorer 162 (20.0%), middle 163 (20.1%), richer 161 (19.8%), and the richest 162 (20.0%). According to this study, 142 (17.5%) of women reported that they had access to transportation (Table 1).

**Table 1:** socio-demographic demographic characteristics of delivered mothers in northwest Ethiopia, 2020

Variables	Categories	Frequency (N, %)
**Age group of the respondents (years)**	15-24	227(27.9)
	25-34	394(48.6)
	35 and above	190(23.4)
**Occupation of the respondents**	Housewife	801(98.8)
	Others (merchant, employee and daily laborer)	10(1.2)
**Maternal education**	No formal education	500(61.7)
	Primary education	215(26.5)
	Secondary and above	96(11.8)
**Paternal education**	No formal education	523(64.5)
	Primary education	205(25.3)
	Secondary and above	83(10.2)
**Household wealth status**	Poorest	163(20.1)
	Poorer	162(20.0)
	Middle	163(20.1)
	Richer	161(19.8)
	Richest	162(20.0)

**Source of information and community participation of the study participants**: this study showed that the proportion of women who had received home visits by Health Extension Workers (HEWs) during pregnancy was found to be 361 (44.5%). Women who reported that they had access to mass media exposure were 140 (17.3%). Our study revealed that, of the total 811 mothers, 394 (48.6%) delivered mothers were members of community-based health insurance, and 260 (32.1%) of the mothers were members of the women's development army (Table 2).

**Table 2:** source of information about antenatal care visits and community participation related variables among delivered mothers in northwest Ethiopia, 2020

Variables	Categories	Frequency (N, %)
**Exposure to mass media (TV or radio)**	Yes	140(17.3)
	No	671(82.7)
**Member of community-based health insurance**	Yes	394(48.6)
	No	417(51.4)
**Member of women development army**	Yes	260(32.1)
	No	551(67.9)
**Getting health education during pregnancy**	Yes	493(60.8)
	No	318(39.2)

**Reproductive health characteristics of the study participants**: in this study, of the ANC visited-women, 358 (50.9%) had attended antenatal care visits within 16 weeks of gestational age, and 321 (45.6%) had completed four or more antenatal care visits. Of the women who had received at least one ANC visit, 358 (50.9%) had attended the first ANC visits before 16 weeks of gestational age, whereas 346 (49.1%) had attended the first antenatal care visits after 16 weeks of gestational age. Our study found that 177 (21.8%) of the women were parity one, whereas 276 (34.1%) of the participants had given birth to five or above. Of all the participants, 642 (79.2%) had the intention of reporting their recent pregnancies. According to our findings, 127 (15.7%) of the women had exposure to pregnancy-related complications (Table 3).

**Table 3:** antenatal care visit status and obstetric history among delivered mothers in northwest Ethiopia, 2020

Variables	Categories	Frequency (N, %)
**Have you attended ANC for recent pregnancy (n=811)**	Yes	704(86.8)
	No	107(13.2)
**Number of ANC visits (n=704)**	One time	33(4.7)
	Two times	97(13.8)
	Three times	253(35.9)
	Four and above	321(45.6)
**The current pregnancy was intended (n=811)**	Yes	642(79.2)
	No	169(20.8)
**Parity (n=811)**	1	177(21.8)
	2-4	358(44.1)
	>=5	276(34.1)
**Facility delivery (n=811)**	Yes	508(62.6)
	No	303(37.4)

**PNC utilization**: our study found that 19.85% (95% CI: 20.8-29.4) of the mothers attended postnatal care utilization.

**Factors associated with PNC service utilization**: women who attended secondary and above-education were 2.65 times (AOR =2.65; 95%CI: 1.43, 4.94) more likely to PNC compared to women who did not attend formal education. This study revealed that women gave birth at the facility were 3.99 times (AOR = 3.99; 95%CI: 2.44, 6.55) more likely to attend PNC than their counterparts. Our study also found that women who have membership in women’s association in the community were 1.6 times (AOR = 1.6; 95%CI: 1.07, 2.4) more likely to utilize PNC services compared to women who had no membership in women development association. According to our study, women who had four or more ANC contacts were 1.51 times (AOR = 1.51; 95% CI: 1.03, 2.22) more likely to attend PNC utilization as compared to their counterparts. The odds of attending PNC utilization were 1.77 times higher (AOR = 1.77; 95% CI: 1.21, 2.58) in women who had visited health post and got health education during pregnancy as compared to women who did not visit health posts and received health education. Women who had a birth order of 3 were 2.64 times (AOR = 2.64; 95%CI: 1.21, 5.78) more likely to attend PNC utilization as compared to parity of one (Table 4).

**Table 4:** factors affecting PNC utilization in a rural northwest Ethiopia, 2020

Variables	Category	PNC utilization		COR (95%CI)	AOR (95%CI)
		Yes	No		
**Age group of the respondents (years)**	15-24	49	178	1	1
	25-34	70	324	0.78(0.52, 1.18)	0.63 (0.34, 1.16)
	35 and above	42	148	1.03(0.65, 1.18)	0.69 (0.33, 1.44)
**Media exposure**	Yes	88	284	1.31(0.85, 1.20)	0.93 (0.58, 1.52)
	No	73	366	1	1
**Women education**	No formal education	89	411	1	1
	Primary education	38	177	0.99(.65, 1.51)	1.02 (0.61, 1.72)
	Secondary and above	34	62	2.53(1.57, 4.08)	2.65 (1.43, 4.94)*
**Pregnancy intendedness**	Yes	139	503	1.85(1.14, 3.00)	1.47 (0.54, 3.99)
	No	22	147	1	1
**Place of delivery**	Facility	138	370	4.54(2.84, 7.25)	2.65 (1.43, 4.94) **
	Home	23	280	1	1
**Member of women development army**	Yes	68	192	1.74(1.22, 2.49)	2.65 (1.43, 4.94)*
	No	93	458	1	1
**Member of health insurance**	Yes	81	313	1.09(0.77, 1.54)	0.80 (0.55, 1.19)
	No	80	337	1	1
**ANC4 and above**	Yes	86	235	2.03(1.43, 2.87)	2.65 (1.43, 4.94) *
	No	75	415	1	1
**Getting education from health post**	Yes	75	239	1.49(1.06, 2.13)	2.65 (1.43, 4.94) *
	No	86	411	1	1
**Planned pregnancy**	Yes	136	499	1.65(1.04, 2.62)	1.08 (0.41, 2.83)
	No	25	151	1	1
**Parity**	1	34	134	1	1
	2	50	308	0.51(0.32, 0.79)	0.99 (0.53, 1.86)
	3 and above	68	208	1.02(0.66, 1.58)	2.65 (1.43, 4.94) *

* Significant at p<0.05; **significant at p<0.00

## Discussion

Variables like women's education, place of delivery, membership in the women's development army, ANC visit, getting health education from the health post, and parity were statistically significantly associated with postnatal care utilization in our study. This study revealed that the odds of PNC service utilization among women who had secondary and higher educational levels were 2.65 times more likely as compared to those who had no formal education. This finding is consistent with studies conducted in Ethiopia [15,16,25-28], elsewhere in Kenya [29], Tanzania [30], and Pakistan [31]. It is for these reasons that education improves the best use of health information on PNC services. In addition, education increases the opportunity for women's engagement in different social and economic activities, which exposes them to social interaction and create the capacity to use PNC services better than those of women who have no education. Furthermore, this may be due to the fact that education enhances female autonomy to develop the capability to make decisions about their health. In the current study, the odds of postnatal care utilization among women who give birth in a health institution were 2.65 times higher as compared to women who deliver their babies at home. This finding is in line with the studies done in Ethiopia [15,16,25,32-34], a study of 36 SSA countries [6], and from 23 countries across Africa, southeast Asia, the eastern Mediterranean, Europe, the Americas, and the western Pacific [13]. This could be explained by the fact that as mothers gets delivered at the health facilities; they are more likely to receive counselling on postnatal care services and danger signs, as well as being exposed to health education.

In this study, we found that women's membership in the development army was significantly associated with PNC among women. Those women who are members of the women's development army were 2.65 times more likely to receive PNC services as compared to their counterparts. This finding is supported by studies conducted in Ethiopia [35,36]. The possible reason might be that participation in groups helps to mobilize and educate the community on PNC with their respective members. Additionally, engagement in the Washington Harbor District Alliance (WHDA) increases women's health seeking behaviour towards PNC service. Furthermore, women's development teams are also used as a main source of information for mothers to prepare themselves for MCH care services. However, a contrasting finding was reported from a study done in Ethiopia [37]. In our study, the odds of postnatal care utilization were higher for women who had a history of at least four antenatal care follow-up services as compared to those who had no four ANC follow-ups. This finding is in line with a number of studies [27,34,38-42]. Results from a pooled analysis of data from 23 countries also support our finding [13]. This is because ANC is the time when women are counselled on danger signs of pregnancy and birth preparedness by skilled healthcare providers, which encourages women to follow postnatal care services immediately. Additionally, women who seek ANC previously show positive healthcare-seeking behaviour, which might be reflected throughout the entire continuum of maternal healthcare. Getting an education from health posts had shown an association with PNC service use in our study. Those women who had contact with health posts were at higher odds of receiving PNC services compared to those had not gotten an education from the health posts. This finding is supported by the studies conducted in Ethiopia where those households who are graduated from health posts by improving their knowledge and implementing health education packages from health posts were more likely to receive PNC services [12,36]. This is because of the improvement of knowledge, skills, and behavior change in health practices due to education of the health post workers would influence the uptake of PNC services.

In the current study, we found that parity was statistically significantly associated with the immediate use of postnatal care services. Those women who had 3 and above parity were 2.65 times more likely to use PNC services as compared to those who had only one. Supportive findings were reported from the studies conducted in Ethiopia [38], and elsewhere in Nigeria [43]. This can partly be explained by the fact that previous experience with pregnancy-related healthcare services may increase the likelihood of seeking postnatal care. On the other hand, contrasting findings were reported from a study done in Ethiopia, where women with low parity were more likely to use PNC services [44], and also from a study conducted in Nigeria [42]. It is possibly related to the fact that those high-parity women do not consider postnatal care valuable based on the experience they gather from previous childbirth. In this study, we assessed community-level postnatal care utilization and factors associated with it using both community- and individual-level variables. The authors have conducted rigorous sample size estimation and applied simple random sampling techniques to reach out to the study participants. Moreover, the adequate training and close supervision provided during the study period were an added value. Recall bias may be the limitation of this study, as the participants might not remember the previous event other than recent events. Nevertheless, we attempted to specify questions related to the service given during the postnatal period by probing. Finally, we had not considered community and facility-level factors in the current study, which could have an impact on the individual factors associated with this study.

## Conclusion

Postnatal care utilization was low in the northwest of Ethiopia. The findings suggest that completing secondary and above education, women health clubs in the community, health education at the health post-level, facility delivery, ANC visit of four or more, and parity or three and above were predictors of PNC utilization. Therefore, the existing health system should consider multilevel intervention strategies focusing on maternal health education, promoting health education at the health post-level, strengthening of women's participation at women development army, and ensuring physical accessibility of healthcare to improve postnatal care utilization in rural Ethiopia.

### What is known about this topic

- *A large proportion of maternal deaths occur during the first 48 hours after birth;*
- *Thus, receiving PNC from a healthcare provider at the recommended time with its appropriate contents also prevents complications that could arise after childbirth;*
- *In Ethiopia, about 71% of rural women do not receive a postnatal check-up within 2 days of delivery, whereas 52% of urban women do not receive an early postnatal check-up.*

### What this study adds

- *Almost eight out of ten women do not receive postnatal care in rural Ethiopia;*
- *Thus, receiving PNC from a healthcare provider at the recommended time with its appropriate contents also prevents complications that could arise after childbirth.*
